# Feasibility of carbon foam-based sorbents for the abatement of gaseous mercury and iodine

**DOI:** 10.1039/d5ra05518k

**Published:** 2025-09-10

**Authors:** Karthikeyan Baskaran, Laurel Sharpless, Casey Elliott, Sean Sullivan, Mackenzie Edinger, Brian Riley, Krista Carlson

**Affiliations:** a Chemical and Materials Engineering, University of Nevada Reno Reno Nevada 89557 USA kc@unr.edu; b Pacific Northwest National Laboratory Richland Washington 99354 USA

## Abstract

The U.S. Department of Energy Hanford Site in Washington State is in the process of commissioning the Waste Treatment and Immobilization Plant to process a portion of the 54 million gallons of radioactive and chemical waste from cold war weapon production. Technologies for the capture of volatile species of concern are still being assessed, and new methods and materials are developed as operational and flowsheet mission risks are identified. One such area still being assessed is the abatement efficacy of the Carbon Adsorber units to retain gaseous mercury and ^129^I released during processing. It is challenging to predict the mercury chemistry due to the variability of the feed, and different methods/materials are required for the capture of gaseous Hg^0^ and Hg^II^ compounds. In this study, the feasibility of using developmental carbon foam (CF) sorbents for the capture of iodine and mercury was assessed using static and dynamic flow testing and compared against a commercially available sorbent, BATII-37. Both CF and CF functionalized with bismuth particles (CF-Bi) chemisorbed iodine, and CF-Bi had similar mercury capture performance to BATII-37 in dynamic flow tests. While species loading concentrations were measurable, limitations in achieving a mass balance prevented a full evaluation of capture efficacy. Nonetheless, the results serve as an important first step in demonstrating the potential for simultaneous iodine and mercury capture.

## Abstract 2

Carbon foam sorbents captured iodine and mercury effectively, showing promise for emission control at Hanford and other industrial facilities.

## Graphical abstract

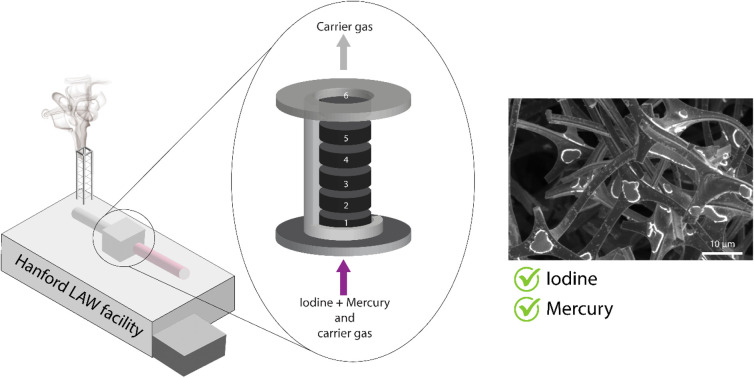

## 1. Introduction

Approximately 56 million gallons of radioactive and chemical wastes produced during the development of nuclear weapons are currently stored at the Hanford Site in Washington State.^1^ The U.S. Department of Energy Hanford Field Office has been tasked with overseeing the safe cleanup of this waste. Washington State has approved the incorporation of this waste into a stable glassy matrix (*i.e.*, vitrification) that is suitable for long-term storage and disposal; however, other materials, such as grout, could be used as long as the waste form meets disposal criteria.^2,3^ The waste is separated into high-level waste (HLW) and low-activity waste (LAW). The HLW fraction is primarily sludge and salt cake, which contains the majority of the long-lived transuranics. The LAW is the supernatant produced during the waste separation process and constitutes about 90% of the tank waste by volume.^4,5^

**Fig. 1:**
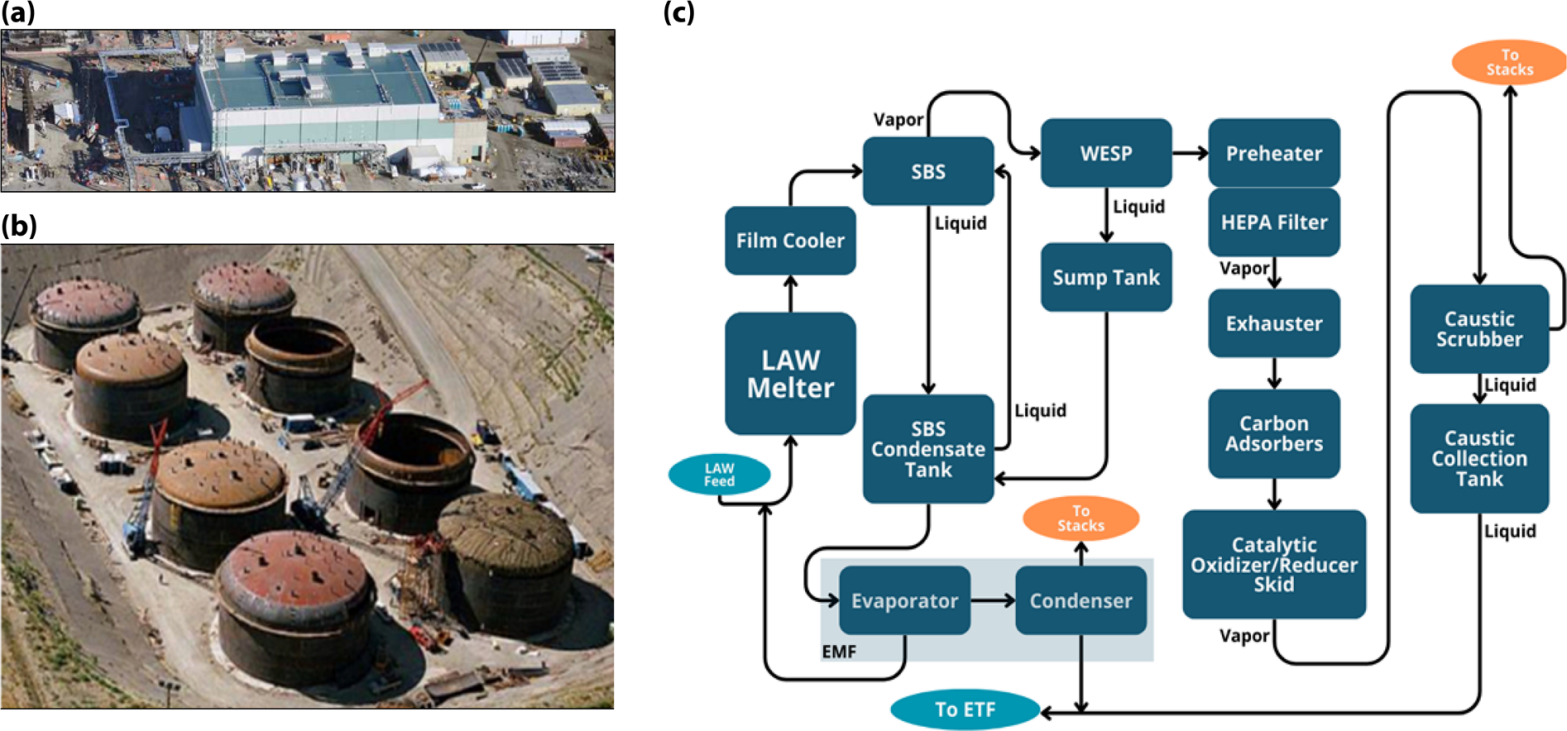
Image of the (a) WTP facility (b) tank farm (with permission from Marcial *et al.*^7^) and (c) the flow diagram illustrates the treatment process the vitrification effluent is expected to undergo (recreated with permission from Fountain *et al.*^10^).

Vitrification operations of HLW and LAW will take place onsite in the Waste Treatment and Immobilization Plant Project (WTP).^1,2^ Eventually, HLW and LAW will be treated simultaneously; however, as challenges still surround the vitrification process for HLW, the LAW will be treated first to enable treatment as soon as possible. Construction of the LAW Vitrification Facility (Fig. 1a) was completed in June of 2021 (ref. 6) and was designed to process waste directly from the tank farms (Fig. 1b).^7^ Glassy waste forms will be produced using two 300 ton melters (20 ft × 30 ft × 16 ft high) that will operate at ∼1150 °C.^8^ In 2023, one of the melters was used to pour a test glass (containing no chemical simulants or radioactive waste) into a large stainless-steel storage container designed to contain the vitrified waste.^9^ Staged filtration, already implanted in the carbon adsorber units (Fig. 1) with primary and guard beds, is likely needed to achieve the desired decontamination factors.

Due to the nature of the high temperature LAW vitrification process, hazardous and radioactive contaminants will be off-gassed, including ^129^I, Hg, NO_*x*_, and acids (*e.g.*, HCl, HF), and have a relatively high humidity.^11^ A complete list with approximate values is provided in Tables S1 and S2 in SI. Fig. 1c is a simplified block flow diagram of the WTP LAW melter off-gas treatment process.^12^ As the off gas leaves the LAW melter, it passes through several stages that pre-treat the effluent and reduce particulate matter. The gas stream first enters a film cooler to reduce solids deposition and cools the stream to ∼315 °C.^7,12^ This is followed by the submerged bed scrubber (SBS) that further cools the gas stream to condense water vapor and remove larger particulate (1–5 μm). The stream then enters the wet electrostatic precipitator (WESP) that removes submicron particulate by ionization, and then into, high efficiency particulate filters (HEPA), which removes 99.95% of any remaining particulates.^7^ The off-gas stream then passes through the carbon adsorber units, whose primary purpose is to reduce the mercury concentration to below 45 μg dscm^−1^.^13^ Additionally, acid gases (*e.g.*, NO_*x*_, HCl, HF, SO_2_, CO_2_) and radioiodine (^129^I) should be removed to <97% and <99.9%, respectively, based on Best Available Control Technology for Toxics (T-BACT) guidelines issued in 2024.^14^ To achieve this, a catalytic oxidizer/reducer skid is used to remove NO_*x*_ and volatile organic carbons (VOCs). Finally, the off-gas stream passes through a final caustic scrubber to remove acid gases before being released to the environment.

The carbon adsorber units currently contain BAT-37, a mixture of 70 vol% sulfur-impregnated (9 wt%) activated carbon and 30 vol% aluminosilicate zeolite, both of which are in granular forms.^10^ The BAT-37 currently in the carbon adsorber units is the last of the inventory from the vendor, and there is no equivalent replacement material currently specified.^12^ An active search for alternatives to BAT-37 is currently underway. One potential alternative sorbent under consideration by WTP that is commercially available is BATII-37, that is the same mixture but with ∼2 wt% sulfur (instead of ∼9 wt% for BAT-37). However, the use of a new sorbent could also address some of the other issues related to the use of activated carbons. For example, activated carbon is friable, creating combustible dusts and particulates that release hazardous and radioactive species contained within them.^15,16^ Combined with the knowledge that iodine is typically physisorbed on the carbon, there may be unintentional releases into the environment when the plant is idling, *i.e.*, it is not in capture mode but is at operating temperature.

In our previous work,^17^ we described a robust (*i.e.*, non-friable) carbon foam substrate (CF) that was developed by carbonizing a melamine foam. Bismuth nanoparticles were electrodeposited onto the CF for the chemical capture of iodine. In this study, we observed that some iodine was chemisorbed by the CF not containing bismuth as a compound identified as C_2_I_4_. This finding prompted further exploration of the CF structure because this result was unexpected as carbon-based sorbents typically capture iodine through physisorption.^18,19^

The presence of NO_2_ in the gas stream can influence both the stability of carbon sorbents,^20,21^ as well as the capture of iodine^22^ and mercury compounds.^23–25^ For iodine capture, metal-based sorbents responsible for chemisorption may become oxidized, thereby reducing the capture efficacy.^22^ For mercury, NO_*x*_ can enhance of inhibit capture depending on the sorbent type and gas composition, through mechanisms such as oxidation of active sulfur sites, reduction of chemisorbed Hg species, or competitive adsorption on carbon surfaces.^23–25^

In this work, we explored the mechanism of iodine capture on the as-formed CF and the feasibility of simultaneous mercury and iodine capture. The capture of iodine and mercury with no additional chemical modification would simplify the fabrication of the sorbent and significantly reduce the fabrication cost. However, bismuth functionalized carbon foam (CF-Bi) was also investigated because chemisorption of iodine through the formation of non-water-soluble compounds is likely needed in the WTP off gas. Challenges related to the accurate quantification of mercury are also discussed to assist those in need of consistent mercury generation for bench-scale testing.

## 2. Methods

### 2.1. Sample preparation and characterization

Commercially available melamine foam (MeF, Basotech^®^) is the precursor material for the carbon foam (CF). The safety data sheet (SDS) states that MeF primarily consists of formaldehyde–melamine–sodium–bisulfite,^26^ but the exact composition and fabrication process are proprietary. The MeF was heated at 10 °C min^−1^ to 900 °C under N_2_ (UHP) in an atmosphere-controlled furnace (Fig. S1). After a 30 min isothermal hold at the terminal temperature, the furnace was shut off and allowed to cool to room temperature before removal. A post-synthesis treatment with NO_2_ was conducted on the CF (referred to as CF-NO_2_) to provide additional insight into the iodine capture mechanism, as discussed in later sections. For this treatment, the CF was exposed to flowing 1 v/v% NO_2_ for 24 h at 150 °C (Fig. S2).

The CF was functionalized with bismuth nanoparticles using a procedure based on previously published work^27^ with modification for enhanced bismuth loading and control over particle size. In this procedure, 1.5 g of bismuth nitrate pentahydrate (Bi(NO_3_)_3_·5H_2_O) was dissolved in 15 mL of HNO_3_. The bismuth solution was poured into a 100 mL Teflon liner along with 35 mL of ethylene glycol to make a total reactant volume of 50 mL. Carbon foam was submerged in the reactant solution and the Teflon liner was inserted into a stainless-steel vessel. The reaction was carried out for 12 h at 180 °C with the heating rate maintained below 10 °C min^−1^. After the completion of the reaction, the vessel was allowed to cool naturally to room temperature and the bismuth-functionalized carbon foam (CF-Bi) was removed and cleaned to remove unwanted contaminants and loosely bound Bi nanoparticles. The cleaning procedure involved ultrasonication of the CF-Bi samples in an isopropanol solution, followed by drying at 100 °C in a vacuum oven for 24 h.

### 2.2. Static testing of iodine and mercury sorption

The CF was statically loaded with iodine in a saturated iodine–air environment to assess maximum iodine adsorption. The initial mass of the CF samples (*m*_s_) was recorded and then loaded onto perforated perfluoroalkoxy alkane (PFA) stands (Savillex). A glass vial containing 1 g of solid iodine was placed alongside the CF samples in a 1 L PFA jar with a PFA lid (Savillex). The PFA jar was then placed in an oven preheated to 150 ± 2 °C for 24 h. The amount of iodine was deliberately chosen to exceed the total foam mass to ensure a saturated vapor environment during sorption, as evidenced by a purple plume existing the container when it was opened within a fume hood. The static loading of mercury was conducted in the same manner but at 71 ± 2 °C.

The temperature for dynamic sorption was selected based on the LAW facility where, off-gas enters the carbon adsorber units at 71 °C, that was chosen to effectively handle technetium and iodine^28^ while minimizing the effect of corrosion due to condensation of acidic species.^29^ However, the static loading reported in this study was conducted at 150 °C to enable comparison with our previously reported results,^17,30^ which were designed to match the conditions in spent nuclear fuel reprocessing.^31–33^ Additionally, static loading of iodine at 71 °C for CF along with other samples was already conducted in our prior study.^34^ The static loading of mercury was only performed at 71 °C because this is the temperature relevant to the LAW off-gas.^17,30^

Following the reaction, the foams were returned to the oven uncovered (with iodine removed) for 1 h at the same temperature to remove loosely bound (physisorbed) iodine, allowing comparison with literature values.^35–37^ The foams were then weighed, returned to the oven uncovered for an additional 24 h, and weighed again to study the stability of captured iodine species. The net mass gain, recorded as *m*_i_, represents the amount of mass gained after the desorption steps (which is attributed to iodine, but could also include hydration). The iodine sorption capacity *Q*_e_ (mg of iodine per gram of sorbent) was calculated using eqn (1). For samples exposed to NO_2_, *m*_s_ corresponds to the mass post-NO_2_ treatment. In addition to static iodine loading, the CF was also tested for mercury adsorption using a similar procedure, but with the temperature set to 71 °C, the temperature relevant to the LAW off-gas.1
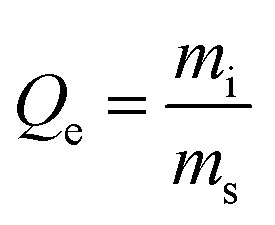


Static desorption was measured at 1 h and 24 h to minimize contributions from physiosorbed iodine. For these sorbents, 1 h was shown to be insufficient for desorption and after 24 h desorption had reached a practical plateau.^17^ Therefore, based on the pore structure and composition of the sorbents, the 24 h values are interpreted as chemisorbed iodine, and no kinetics parameters are inferred from these measurements.

### 2.3. Dynamic flow testing of iodine and mercury sorption

**Fig. 2:**
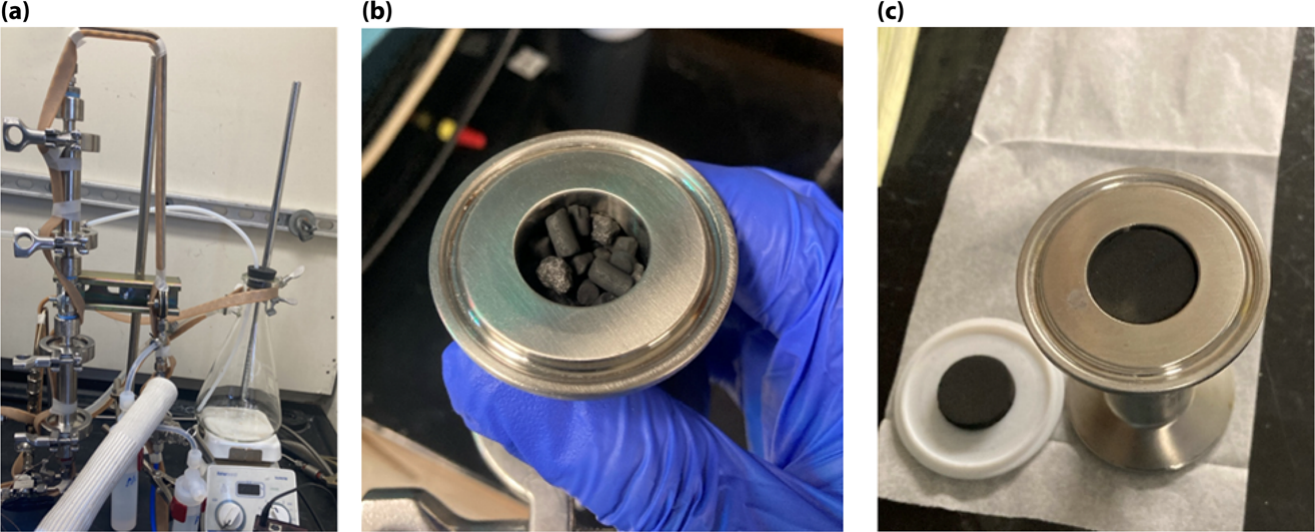
(a) The V2 system used for dynamic flow testing of iodine and mercury. The 316 stainless steel bed loaded with (b) BATII-37 and (c) carbon foam.

An overview of the methods related to the dynamic testing is provided here, and details are provided in the SI. Iodine and mercury were introduced into ambient air using a Dynacalibrator (Dynacal, Metronics Inc., 230-28B-I). The air was then mixed with other components (*e.g.*, water vapor, NO_2_, and NO), heated to 71 °C, and passed through stainless steel cylindrical sorbent beds (0.87 inch inner diameter and 3 inch length), and into a PTFE submerged bed scrubber containing *aqua regia*. The additional water vapor ranged between 1–5 mass% and NO_*x*_ was ∼0.5 v/v%. Two different flow set ups were used for data collection. The V1 system (Fig. S9) was used for initial testing on the carbon foams (CF-1 through CF-6). Due to supply chain issues, the sorbent bed was 304 stainless steel. A simplified V2 system (Fig. 2a) was designed and constructed after challenges controlling the gas flow through the V1 system. This system had all 316 stainless steel parts and was used to collect data for carbon foams functionalized with bismuth (CF-Bi) and BATII-37. The use of 316 stainless steel for the infrastructure instead of a non-interactive surface (*e.g.*, polytetrafluoroethylene, PFA) was to simulate the LAW off-gas infrastructure, as this could greatly impact mercury speciation and capture efficacy. Oxidizing reactions with hydrogen halide acid gases (*e.g.*, HI, HCl) may not occur to the same extent in the absence of reactive surface sites, and therefore, lead to inflated (inaccurate) capture values.

The CF-based sorbents were made into disks (0.9 inch diameter and ∼0.75 inch height) before loading into the chambers (Fig. 2b). For each exposure, six disks were loaded into the bed, labeled 1 through 6, with disk 1 located closest to the inlet. The mass of the sorbent loaded into the chamber depended on the sorbent density, *i.e.*, ∼20 g BATII-37 (granular media), ∼0.25 g of CF, or ∼1 g of CF-Bi. Sorbent loading was semi-quantitatively analyzed using EDS analysis. Mercury loading was quantified by acid leaching the sorbents with aqua regia on an orbital shaker for ≥16 hours. The leachate was then preserved using BrCl according to EPA Method 1631 (Revision E).^38^ Aliquots of the preserved leachate were tested using a cold vapor atomic fluorescence spectroscopy (CVAFS) according to EPA Method 1631 (Revision E) (Tekran 2600-IVS).

### 2.4. Sorbent characterization

Thermogravimetric analysis (TGA, Netzsch STA 449F3) was performed on the MeF at 10 °C min^−1^ to 900 °C to evaluate weight loss during conversion to CF. Scanning electron microscopy (SEM, Thermo Scientific Scios 2) with energy dispersive X-ray spectroscopy (EDS, EDAX Octane) and transmission electron microscopy (TEM, JEOL JEM 2800) were used to examine the physical and chemical properties of the sorbents. Raman (Thermo Scientific DXR) and Fourier transform infrared (FTIR, Thermo Scientific Nicolet 380) spectroscopy were performed to evaluate the extent of carbonization and structural changes after NO_2_ treatment and iodine adsorption. The relative intensity of graphite peak in the Raman spectrum was used as a parameter to check the extent of carbonization. X-ray diffraction (Rigaku Smartlab, with Cu source) was used to determine crystalline phases. The surface area of the sorbents were calculated using Brunauer–Emmett–Teller (BET) theory using argon gas (Anton Paar, Autosorb 6300 with CryoSync control).

## 3. Results and discussion

### 3.1. Properties of CF

**Fig. 3:**
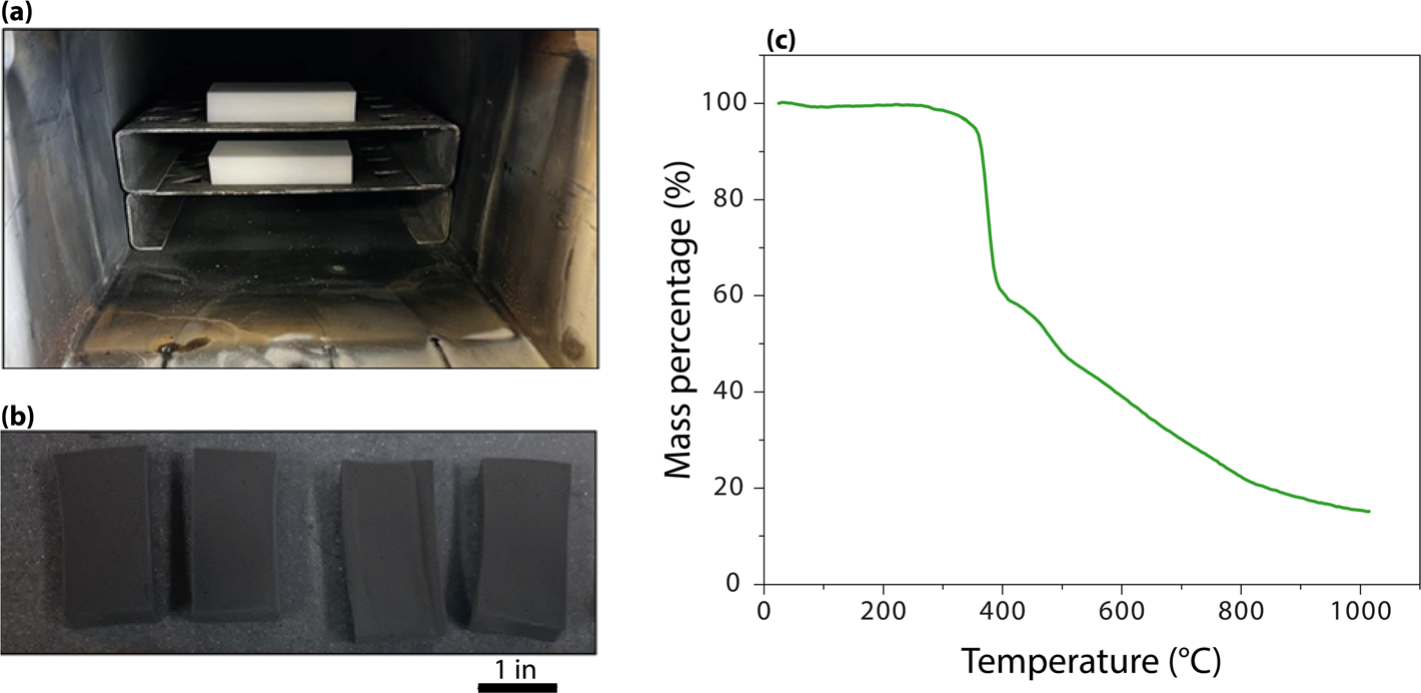
(a) Picture of MeF loaded in the furnace, (b) picture of CF formed from carbonization, and (c) TGA of MeF showing mass loss with temperature in N_2_.

**Fig. 4:**
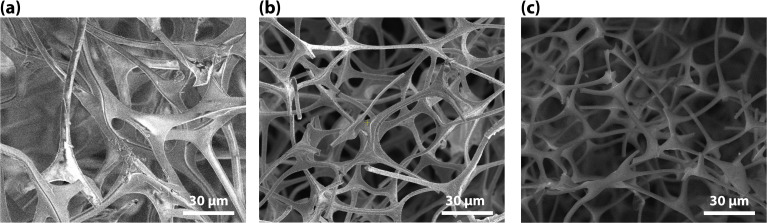
SEM micrographs of (a) melamine foam (MeF), (b) carbon foam (CF), and (c) carbon foam after NO_2_ treatment (CF-NO_2_).

**Fig. 5:**
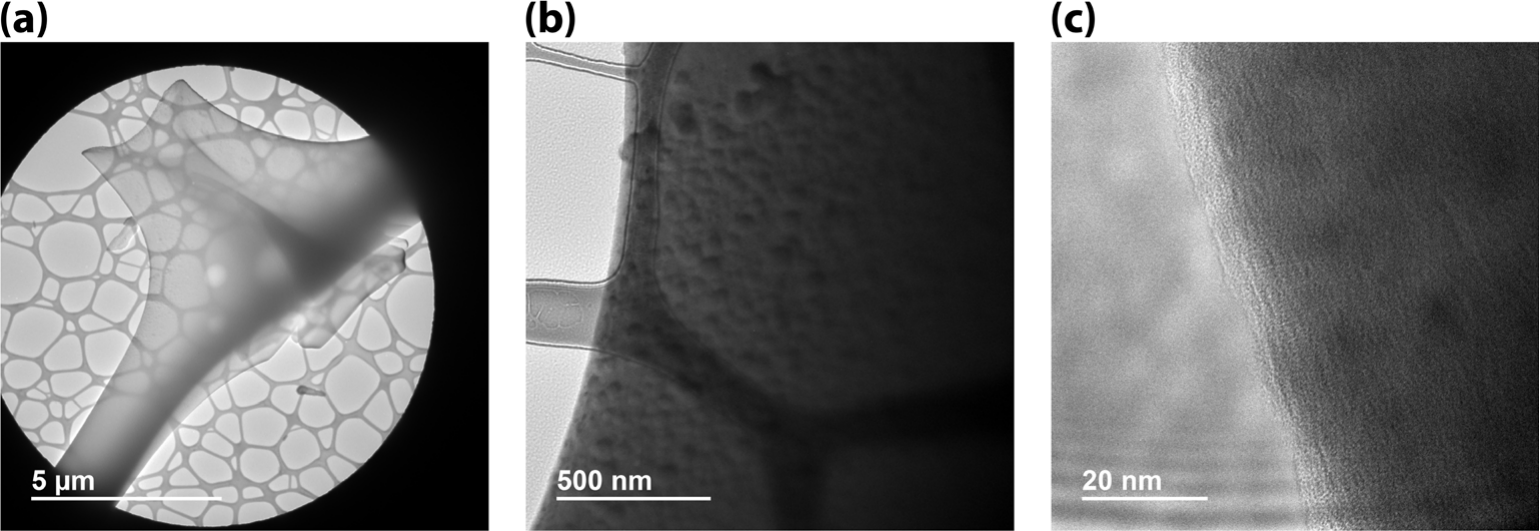
(a) TEM micrograph of CF, (b) higher resolution showing pores, and (c) amorphous structure.

The white, as-received MeF had a ∼50% reduction in volume and ∼80 mass% loss during carbonization (Fig. 3a and b). The interconnectivity of the framework did not appear to change significantly with carbonization, although a small, statically significant change in the strand size was observed with microscopy. The MeF strand diameter of 3.15 ± 0.95 μm reduces to 1.99 ± 0.20 μm upon conversion to CF (Fig. 4a and b). TEM micrographs (Fig. 5) of the CF showed pores between 30–70 nm and an amorphous structure.

EDS detected sulfur in the MeF and sodium in the MeF and CF (Fig. S3). While the composition of MeF from different manufacturers appears to be similar based on the safety data sheets, some variations exist in the exact compositions from sample to sample, and the exact chemistry and formation processes for each are proprietary. Interestingly, the MeF first reported by our group did not contain sodium.^17^

**Fig. 6:**
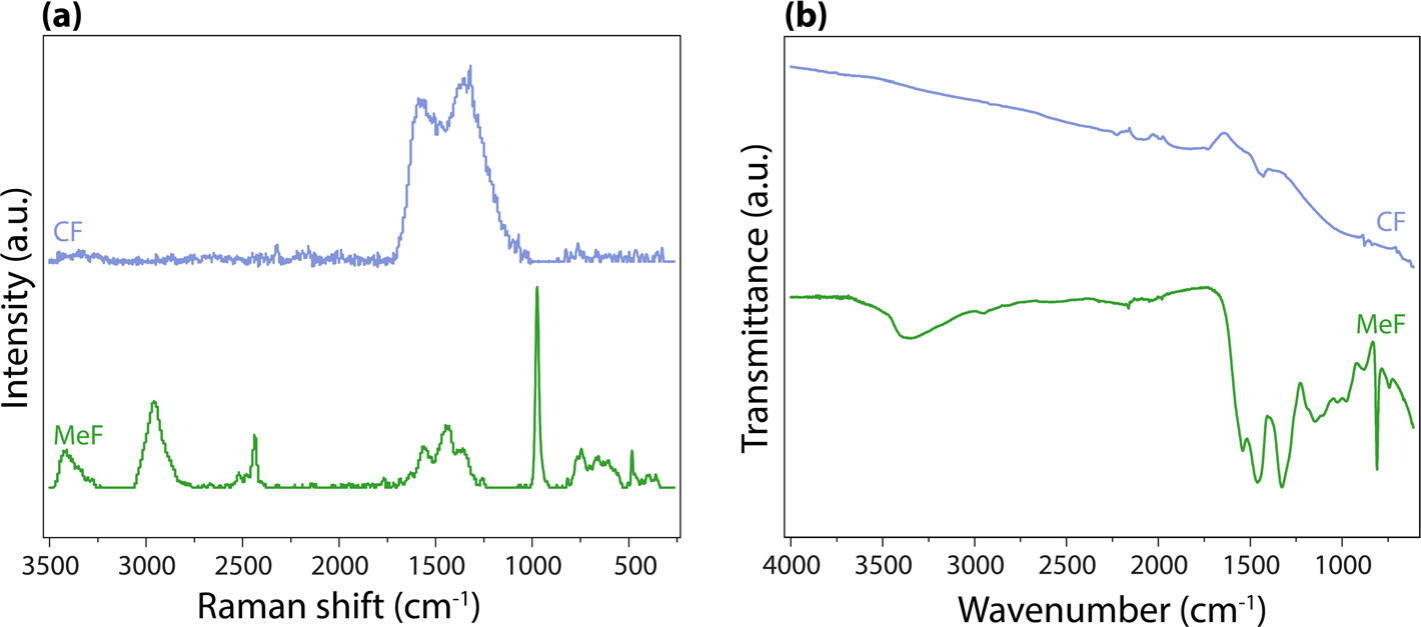
(a) Raman and (b) FTIR spectroscopy of MeF and CF.

Raman and FTIR spectra show structural changes between MeF and CF (Fig. 6). The Raman spectrum of MeF shows bands corresponding to the triazene ring at 973 cm^−1^; carbon–carbon bonds between 600–800 cm^−1^; carbon–hydrogen bonds at 1375 cm^−1^, 1440 cm^−1^, and 2900 cm^−1^; carbon–nitrogen bonds at 760 cm^−1^ and 1560 cm^−1^; nitrogen–hydrogen bonds at 2400 cm^−1^; and hydroxyl bonds at 3420 cm^−1^.^26,39^ Raman spectroscopy of CF showed bands associated with diamond (D) at ∼1330 cm^−1^ and graphite (G) at ∼1580 cm^−1^ with an *I*(D)/*I*(G) peak intensity ratio of 1.22. CF with ratios between 1.21–1.23 suggest near complete carbonization when treated in a nitrogen environment between 800 °C and 1000 °C.^26^ The FTIR spectrum of the MeF shows the presence of the triazene ring at 1533 cm^−1^, 1460 cm^−1^, 1320 cm^−1^, and 813 cm^−1^, and NH_3_ at 3400 cm^−1^.^40,41^ After carbonization, carbon bonds at 2230 cm^−1^ appeared predominantly along with a carbon–OH bond at 1430 cm^−1^, while the peaks associated with triazene and NH_3_ were absent. Lists of peaks for Raman and FTIR are provided in Tables 1 and 2.

**Table 1:** Raman spectrum with peaks from MeF and CF

Raman shift (cm^−1^)	Band assignments	Sample	Ref.
600	C–C	MeF	26 and 39
800	C–C	MeF	26 and 39
760	C–N bend	MeF	26 and 39
973	H_3_N_3_ (triazene ring)	MeF	26 and 39
1330	D-band	CF	26
1375	CH_3_	MeF	26 and 39
1440	CH_2_ or CH_3_	MeF	26 and 39
1560	CN	MeF	26 and 39
1580	G-band	CF	26
2400	N–H	MeF	26 and 39
2900	CH or CH_3_	MeF	26 and 39
3420	OH	MeF	26 and 39

**Table 2:** FTIR spectrum with peaks from MeF and CF

FTIR spectrum (cm^−1^)	Band assignments	Sample	Ref.
813	Triazene ring	MeF	40 and 41
1320	Triazene ring	MeF	40 and 41
1430	C–OH	CF	26
1460	Triazene ring	MeF	40 and 41
1533	Triazene ring	MeF	40 and 41
2230	C–C	CF	26
3400	NH_3_	MeF	40 and 41

The surface areas of the carbon foam-based sorbents are similar, and two orders of magnitude lower than the BATII-37 (Table 3). The high specific surface area and adsorption isotherm (Fig. S4) indicates that BATII-37 is dominated by micropores, which is consistent with literature.^42–44^ There is no evidence of micropores in carbon foams (Fig. S4). Therefore, adsorption of I and Hg is limited to the surface of the strands, making desorption of physisorbed or weakly chemisorbed species a possibility during operation.^42,43^ For CF-Bi, the presence of Bi on the surface enables chemisorption with I, which would mitigate unplanned release.^45,46^ However, the limited availability of functional groups for Hg adsorption highlights the need for further modification to improve the simultaneous capture of both I and Hg.^47,48^

**Table 3:** Surface area of sorbents calculated using BET theory with argon probe gas at 87.3 K

Sorbent	Surface area (m^2^ g^−1^)
CF	2.426
CF-NO_2_	2.595
CF-Bi	2.607
BATII-37	667.455

### 3.2. Static iodine loading on CF and CF-NO_2_

The structure of the CF-NO_2_ looks like the CF (Fig. 3) with no statistically significant difference in strand size (2.08 ± 0.35 μm). Neither the CF nor the CF-NO_2_ were friable during handling, but CF-NO_2_ had less (qualitative) deformation recovery after compression. From gravimetric analysis, the average static iodine loading of CF appeared to be over twice that of CF-NO_2_ (Table 4). CF lost 53% of its mass after 24 h desorption while CF-NO_2_ lost 85%. The majority of the mass loss is attributed to the release of physiosorbed I_2_ (g). Mass loss could also be attributed to the degradation of iodine complexes formed with the carbon matrix, such as C_2_I_4_ observed in our previous work.^17^ Since mass loss plateaued after 24 h, sorbent characterization was conducted after this time point.

**Table 4:** Gravimetric analysis of iodine loading after 1 h and 24 h desorption

Sample ID	*Q*_e_ after 1 h desorb (mg g^−1^)	*Q*_e_ after 24 h desorb (mg g^−1^)
CF-I_2_	490 ± 70	228 ± 40
CF-NO_2_-I_2_	240 ± 42	34 ± 14

**Fig. 7:**
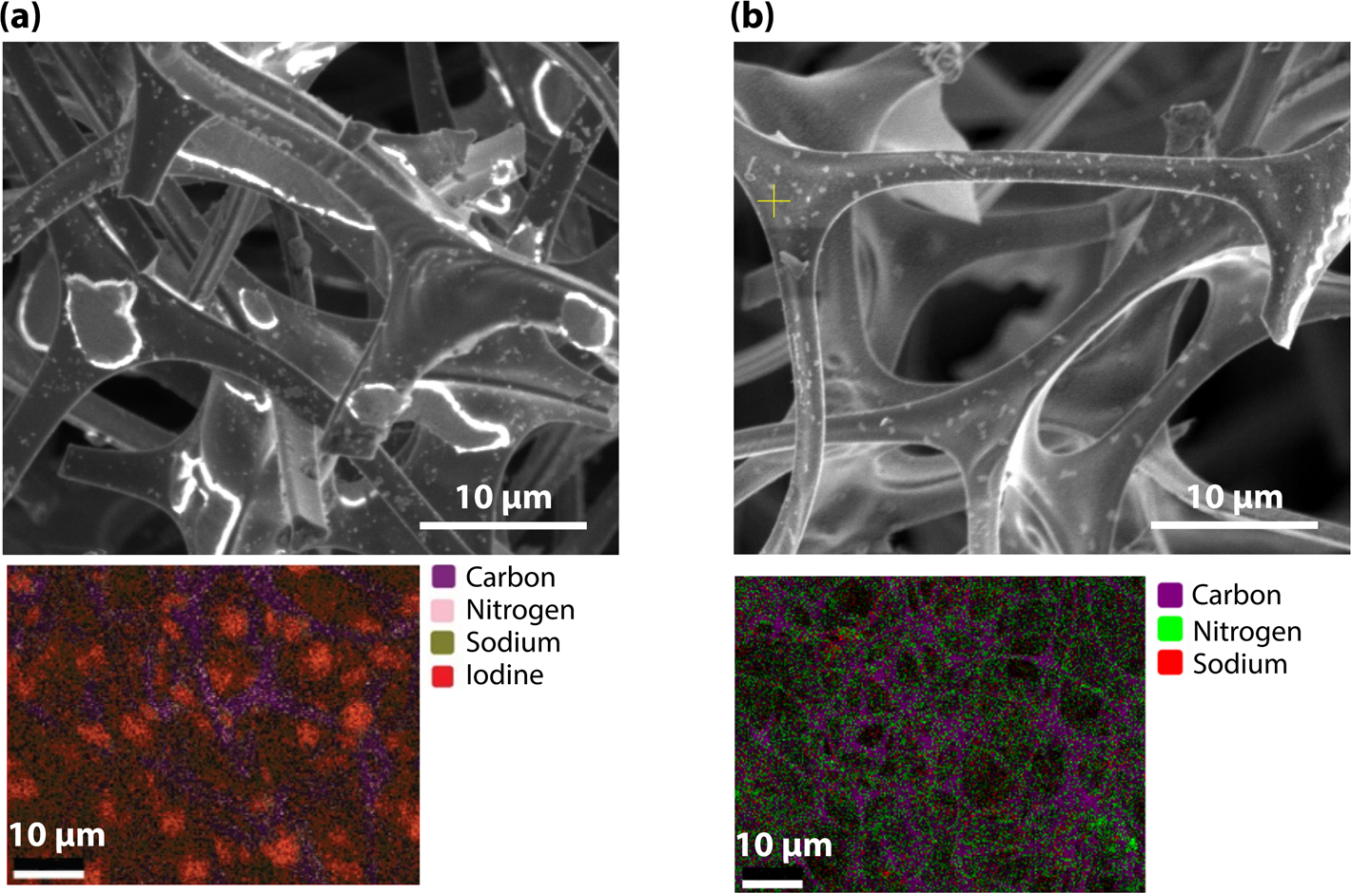
SEM/EDS of (a) CF-I_2_ and (b) CF-NO_2_-I_2_.

Under SEM analysis, ∼4 μm diameter particles were observed on CF loaded with iodine (CF-I_2_), which appeared to be iodine compounds associated with sodium (Fig. 7a). The particles were smaller for the CF-NO_2_-I_2_ (*i.e.*, ≈500 nm in diameter), and did not appear to be chemically distinct from the carbon strand (Fig. 7b). The diffused presence of iodine in both CF-I_2_ and CF-NO_2_-I_2_ further suggests that the CF matrix can strongly bind iodine through physisorption or by forming a complex. The iodine present in CF was water soluble, as no iodine was detected by EDS after a 24 h soak in water followed by drying in an oven at 60 °C for 24 h. EDS analysis (Fig. S5) showed little sodium remaining in the foams.

**Fig. 8:**
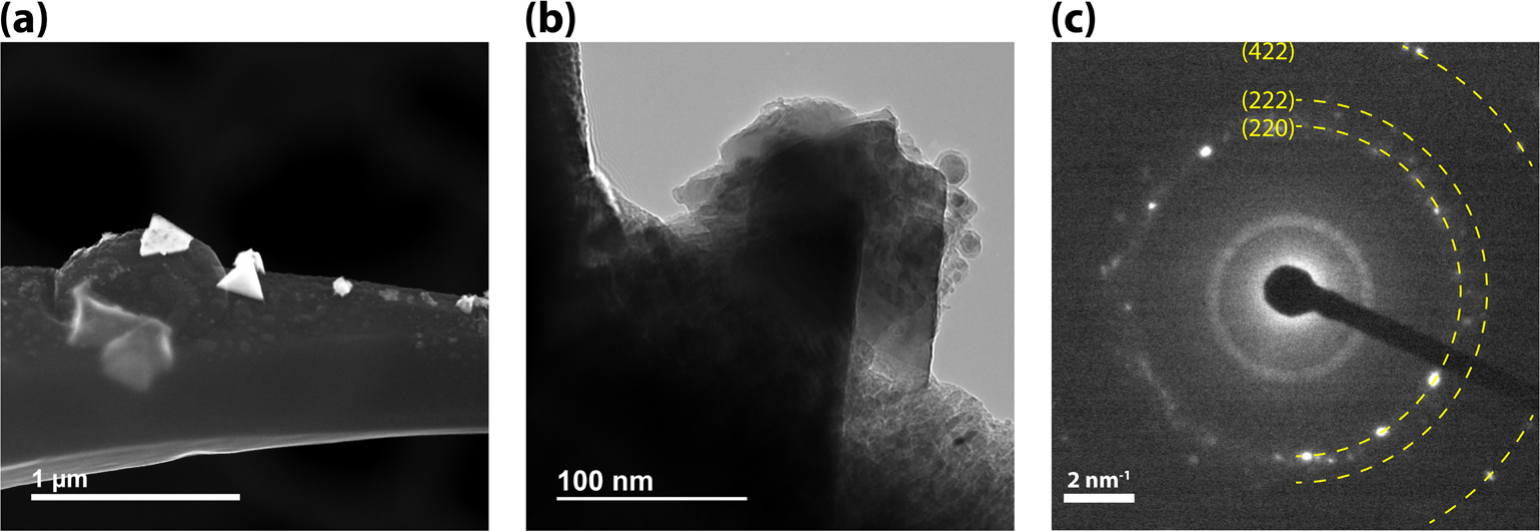
(a) TEM image if CF-I_2_, (b) HR-TEM image of CF-I_2_, and (c) SAED pattern matching NaI from ICDD 00-006-0302.

TEM of CF-I_2_ (Fig. 8) showed similar pores as seen in CF but with higher contrast, suggesting the presence of a heavier element than carbon. Along with the formation of distinct pyramidal shaped particles on the strand, EDS analysis showed diffused presence of iodine through the strand with increased concentration in the particles. These particles were identified as NaI on closer examination with HR TEM mode and selected area electron diffraction (SAED) pattern.

**Fig. 9:**
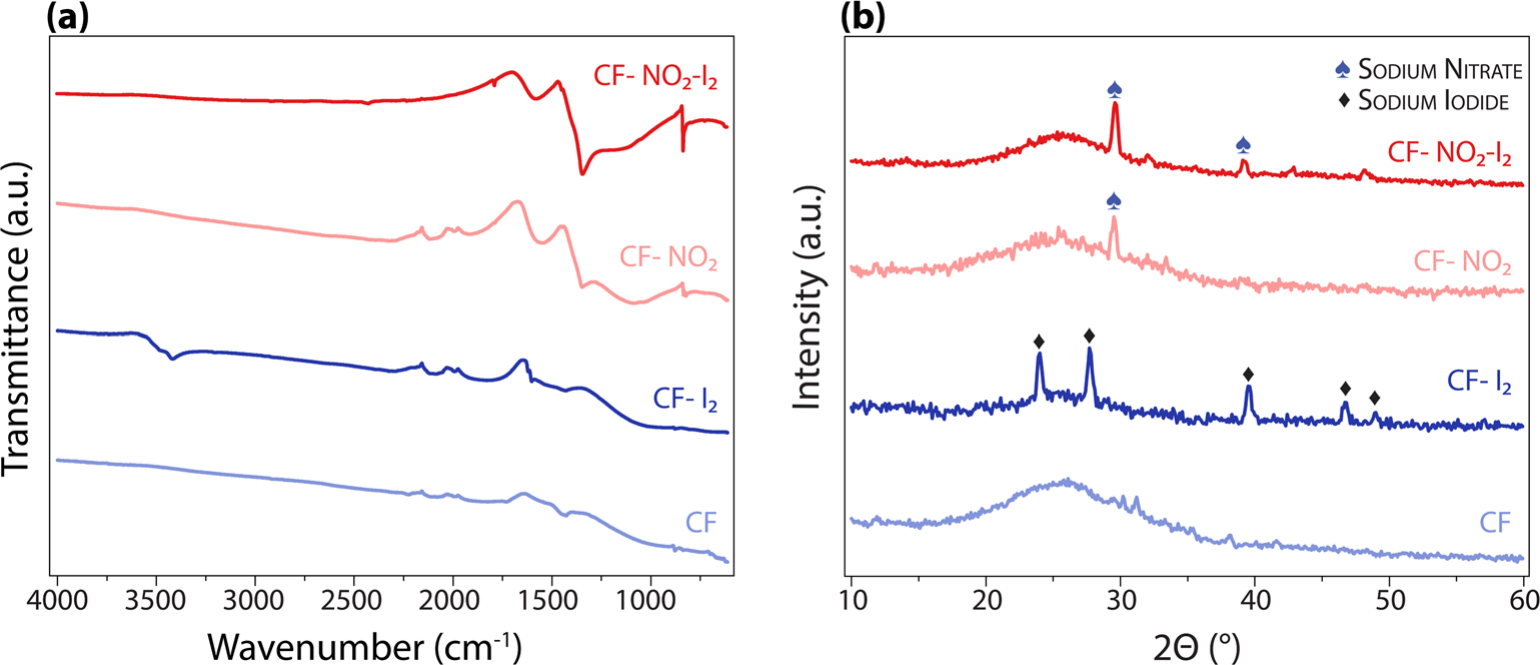
(a) FTIR and (b) XRD of samples showing absence of sodium iodide species after treating with NO_2_.

FTIR spectroscopy of CF-I_2_ showed peaks at 3482 cm^−1^, 3415 cm^−1^, 1632 cm^−1^, and 1604 cm^−1^, all of which are associated with NaI (Fig. 9a).^49^ Peaks corresponding to NaNO_3_ were identified in both CF-NO_2_ and CF-NO_2_-I_2_ spectra at 1788 cm^−1^, 1350 cm^−1^, and 834 cm^−1^ while NaI peaks were no longer observed.^50,51^ The inability of sodium to participate in bonding with iodine was further supported by XRD (Fig. 9b), in which NaI was detected in CF-I_2_ but not in CF-NO_2_-I_2_.

Previous studies conducted at 71 °C showed higher iodine loading; however, these results are likely due to contamination from simultaneous testing of other samples in the same container.^34^ However, EDS confirmed that iodine was present. For static mercury loading, there was no significant change in mass after static loading at 71 °C; however, EDS (Fig. S6) analysis showed mercury present in the carbon foam after 24 h desorption.

### 3.3. Dynamic testing for the capture of mercury and iodine on CF and CF-Bi

The operating parameters and results for the dynamic flow tests are provided in Table 5. Dynamic testing was initially conducted on CF using the V1 system with iodine in an ambient air carrier stream. Exposure times were selected to fully saturate two sorbent beds in series (Run CF-1) and 50% loading of the bed (Run CF-2) based on the static iodine loading capacity of the CF (300 mg g^−1^).^17^ EDS analysis of the disks detected iodine on all disks for CF-1 and only on disks one through three for CF-2. The result for CF-2 showed that the foams load in a linear fashion along the length of the bed, saturating each disk before loading the next in this simplified gas stream.

**Table 5:** Conditions and results from dynamic flow tests. Testing on CF-1 through CF-6 was conducted using the V1 system. All testing on CF-Bi and BATII-37 (labeled as BAT in the table) were conducted using the V2 system

Sample id	Gas stream				Exposure time (h)	EDS analysis (Y or N)	Measured Hg using CVAFS (mg g^−1^)
	I conc. (ppm)	Hg conc. (ppm)	Added H_2_O (Y or N)	NO_*x*_ (Y or N)			
CF-1	0.75	0	N	N	242	I : Y	—
						Hg : N/A	
CF-2	0.75	0	N	N	26	I : Y	—
						Hg : N/A	
CF-3	0.85	0.55	N	N	26	I : N	—
						Hg : Y	
CF-4	0.85	0.55	N	N	24	I : Y	—
						Hg : Y	
CF-5	24.0	2.00	N	N	47	I : N	—
						Hg : Y	
CF-6	0.05	0.59	N	N	24	—	0.8
CF-Bi-1	0.03	0.69	N	N	24	—	30
CF-Bi-2	0.03	0.69	Y	Y	24	—	56
CF-Bi-3	0.03	0.69	Y	Y	24	—	34
BAT-1	0.03	0.69	N	N	24	—	13
BAT-2	0.03	0.69	N	N	24	—	5
BAT-3	0.03	0.69	N	N	24	—	19
BAT-4	0.03	0.69	Y	Y	24	—	24
BAT-5	0.03	0.69	Y	Y	24	—	107

After achieving predictable iodine loading in the dynamic flow system, mercury was introduced into the gas stream. The co-generation of mercury and iodine within the same chamber in the Dynacal creates a favorable condition for the formation of HgI_2_ (g).^52^ At an I : Hg ratio of 1.5 (CF-3 and CF-4), both iodine and mercury were detected on the disks, while at an I : Hg ratio of 0.06 (CF-5 and CF-6), only mercury was detected. These results could indicate the possibility of the capture of both oxidized and elemental mercury. However, the vast majority may be Hg^II^ (s) due to oxidation of Hg^0^ (g) upon interactions with the stainless steel components *via* eqn (2) and (3) shown in Table 6.^53,54^

**Table 6:** Equations for possible mercury reactions in the dynamic flow system.^52^

Reaction equation	Equation
Hg^0^ (g) → Hg^0^ (ads)	(2)
Hg^0^ (ads) + M_*x*_O_*y*_ → HgO + M_*x*_O_*y*−1_	(3)
HCl (g) → HCl (ads)	(4)
2HCl (ads) + O* → 2Cl* + H_2_O	(5)
Hg^0^ (ads) + Cl* → HgCl	(6)
Hg^0^ (g) + Cl* → HgCl (s,g)	(7)
HgCl (s,g) + Cl* → HgCl_2_ (s,g)	(8)

The V1 system was replaced with the simpler V2 system to provide better control over gas flow through the system. Mercury loading on CF, CF-Bi, and BATII-37 was quantified after exposure to I : Hg ratios of 0.4 in ambient air and gas streams containing water vapor and NO_*x*_ in the V2 system (Table 5). EDS spectrum is provided in Fig. S7 in SI. Because a closed mercury mass balance was not achieved, capture efficiencies could not be determined. Therefore, apparent capture capacities (mg g^−1^) were reported. All sorbents had capacities of similar magnitude, but the data should be interpreted in light of the noted mass-balance uncertainty.

The values for the CF-Bi and BATII-37 are similar despite the CF having no sulfur impregnation. Variability in the captured mercury is likely a result of physisorption-based capture, which may also account for the greater loading observed in tests containing H_2_O (g) and NO_*x*_ (g) in the gas stream. The reduction of particulate release when H_2_O (g) is present could have also contributed to the higher loading (see Section 3.4). For CF-Bi it is not clear if these compounds are associated with iodine and bismuth, or the carbon matrix. Further studies are needed to understand the effects of speciation on capture efficacy; however, speciation is an ongoing issue with mercury due to deficiencies in mercury collection and quantification equipment.^10^

### 3.4. Challenges with mercury quantification

**Fig. 10:**
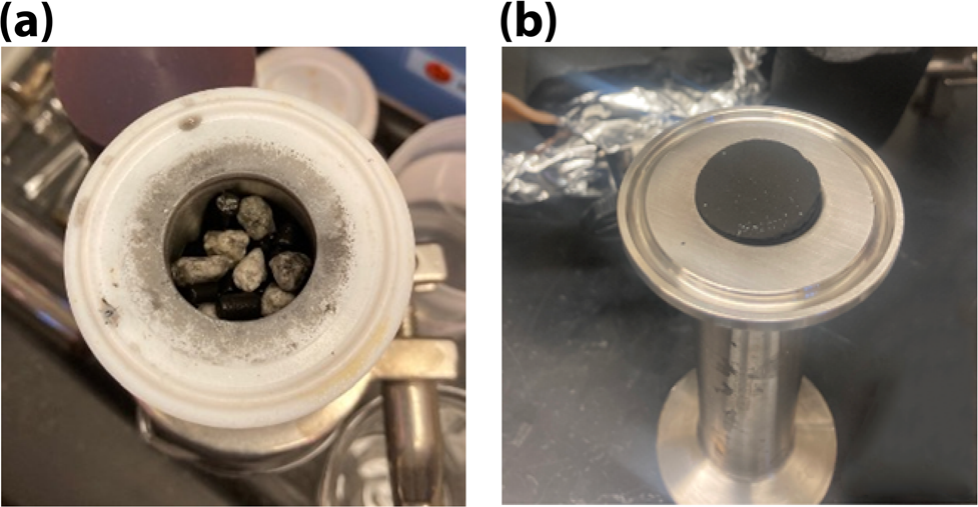
(a) For BATII-37, carbon particulate collected on the flat (non-sealing) surfaces of the flange gasket. This particulate was not observed for the (b) CF or CF-Bi (not shown).

As previously mentioned, the stainless steel components were used in the dynamic flow systems to better simulate reactions that could occur in the LAW off-gas system. To improve control over gas flow, the more complex V1 system was replaced with the simplified V2 design; however, a mass-balance was never achieved, likely due to a combination of mercury species interactions with the Dynacal and dynamic flow system, formation and condensation of HgI_2_ (g), and inability of species to be fully captured by the scrubber solutions due to varied oxidation state. Additionally, for the BATII-37, carbon-particulate would accumulate on the non-sealing surfaces of the flange gasket after each run (Fig. 10a). The photograph of BATII-37 after removing from holder is shown in Fig. S8 in SI. The high friability of the material could also lead to loss. No particulate was observed after the carbon foam tests (Fig. 10b).

Future investigations into mercury–sorbent interactions should include varying ratios of elemental-to-oxidized mercury, as well as potential interactions with the sorbent bed material. Corrosive interactions between gas-phase components and the LAW infrastructure are anticipated. While effects of oxidizing gases and corrosion products on sorbent performance have been acknowledged, the oxidation state of mercury remains a critical factor in determining capture efficacy. Although mercury may be released from the reactor primarily in its elemental form, it can undergo future reactions with reactive species present on containment surfaces or within the effluent.

Iron and chlorine detected on the disks support the formation of Hg^II^ (s) compounds. As mentioned previously, the use of stainless steel for the infrastructure was to provide a more realistic environment in which reactive surface sites could lead to a greater extent of mercury oxidization than expected. On the stainless steel bed surface, chlorine may be present as physiosorbed species or chemisorbed products (*e.g.*, FeCl_3_) formed through reactions with the metal.^55^ The adsorbed chlorine species can react with Hg^0^ (g) to form gaseous and solid oxidized mercury compounds,^56^ which may or may not bind strongly to the sorbent. For example, hydrogen halide acid gases (*e.g.*, HI, HCl) can react with Hg^0^ (g) *via* the following surface-mediated reactions shown in eqn (4)–(9) given in Table 6.^57^

One of the main challenges experienced during testing was determined to be the variability in species release from the Dynacal. While this instrument may be suitable for non-reactive gases, it does not appear to be suitable for species that are highly reactive and/or low condensation temperatures. In the Dynacal (Fig. S10), only the permeation chamber is heated, and many of the critical parts are not Teflon (*e.g.*, the permeation chamber, differential pressure regulators), which can allow for reactions with and/or accumulation of mercury or iodine. It was determined that weeklong bake-out periods were necessary to reduce residual iodine and mercury to below levels that would not interfere with measurements (Fig. S11 and S12). Although reasonably consistent results could be achieved afterward, this approach makes the Dynacal impractical for short-term tests requiring replacement of permeation tubes with different species or rates. As a result, even with a dual-chamber system, a true mass balance would likely not have been achieved. For testing with mercury or iodine, a heated fluoropolymer/glass system is recommended to minimize concentration fluctuations that could be mistaken for sorbent behavior. Given these system limitations, the data collected in this study served as an initial demonstration of capture feasibility.

### 3.5. Sorbent feasibility for capture

Static and dynamic testing demonstrated that CF and CF-Bi have the potential to capture I_2_ (g), Hg^0^ (g), and Hg^II^ (g) compounds. Additional studies are needed to understand mercury speciation in a prototypical LAW off-gas stream, the resulting mechanism of mercury capture, and the stability of the resulting complexes. While sulfur-compounds are effective for capturing elemental mercury, they are not as effective for Hg^II^ (g) compounds. Catalytic oxidation of Hg^0^ (g) with subsequent formation of mercury compounds using oxide-functionalized CF could potentially mitigate issues related to thermal stability and reemission following secondary reactions with other components in the off-gas.

Regarding the CF, iodine either chemically binds with sodium to form NaI (s) or strongly interacts with the carbon-matrix. While these mechanisms provided consistent capture performance in simple air streams, the water-solubility of these phases likely makes as-formed CF an unsuitable iodine sorbent for the high humidity environment of the LAW off-gas stream. To mitigate this risk, functionalization with a metal that forms an insoluble iodide compound upon capture, as demonstrated with CF-Bi, will likely be necessary to enhance iodine retention during operation. While I_2_ (g) chemically reacts with bismuth to form BiI_3_ (s), potential remains for unintended iodine release due to secondary reactions in the off-gas stream. For example, in previous work, we have shown that exposure to NO_2_ leads to the conversion of BiI_3_ (s) to BiOI (s) with subsequent release of iodine.^30^ Direct capture as BiOI (s) using an engineered Bi–Bi_2_O_3_ core–shell structure particles may mitigate transformations that could compromise iodine immobilization. The stability of BiI_3_ and BiOI compounds in a recent study can be used to aid these types of studies.^58^

## 4. Conclusions

Carbon foam-based sorbents successfully captured I_2_ (g) and Hg^II^ (g) compounds from an ambient air carrier stream at loadings comparable the commercial sorbent BATII-37 under similar conditions. Despite the limitations of the experimental setup, the results offer a valuable first step in demonstrating the feasibility of capture, as well as informing future system design and testing protocols. With some additional tailoring for the chemisorption of iodine and various forms of mercury (*e.g.*, Hg^0^, Hg^II^ compounds), the carbon foam-based sorbents may be viable for the carbon adsorber units in the WTP LAW facility at Hanford. While promising, several factors must be considered to fully assess CF-based sorbents under realistic operating conditions.

First, iodine capture on CF occurred through the formation of NaI (s). After exposure to NO_2_ (g), sodium was oxidized to NaNO_3_, effectively eliminating its ability to react with iodine. Despite this passivation, CF still strongly bound iodine throughout the carbon matrix. As both these phases are water soluble, they may not retain the captured iodine in this humid environment, rendering them as effective as physisorption on activated carbon. Therefore, additional functionalization is likely needed to ensure the chemical stability of the captured species in the presence of the off-gas components to minimize the risk of unintentional release during operation or idling.

Second, if bismuth is selected as the reactive metal for iodine chemisorption, engineered bismuth compounds could be used for the direct formation of stable BiOI (s). However, aging in the complex gas stream may naturally lead to these stable phases and binding of any released iodine. Additionally, metal functionalization of the sorbent must not compromise the mechanical robustness, which could lead to particulate release during operation or idling.

Third, the capture mechanism for Hg^II^ compounds is still unclear; however, differences in loading behaviors between tests with different I : Hg molar ratios highlight the importance of speciation on capture. Although the off-gas stream is expected to contain mercury primarily in its elemental form, a significant amount of Hg^II^ compounds will likely also be present due to the acids (*e.g.*, HCl, HF) and oxidants (*e.g.*, NO_*x*_, SO_*x*_) in the off-gas, and interactions with the infrastructure. Therefore, in addition to sulfur-impregnated surfaces for the capture of Hg^0^ (g), surfaces with reactive basic sites will also likely be required for strong binding of Hg^II^ (g) compounds. Staged filtration is likely needed to achieve the desired decontamination factors.

A key limitation of this study is the absence of a closed mercury mass balance across gas and solid phases, which introduces uncertainty into the reported capture capacity. Accordingly, the efficiencies reported here should be interpreted as apparent values that may be biased by interactions with the setup or species conversion, and future work will implement full mass-balance protocols to quantify and reduce the associated uncertainty.

In addition to addressing these factors, further testing under prototypical conditions is needed to evaluate the capture kinetics and loading capacity of various mercury and iodine compounds. These studies will provide a more complete understanding of material performance and decontamination factors, guiding the development and optimization of sorbents suitable for implementation in the WTP, as well as other industries requiring mercury abatement in complex off-gas environments.

## Author contributions

The manuscript was written through contributions of all authors. Karthikeyan Baskaran: conceptualization, investigation, formal analysis, data curation, methodology, writing – original draft, writing – review and editing. Laurel Sharpless: conceptualization, investigation, formal analysis, data curation, methodology, writing – review and editing. Casey Elliott: investigation, formal analysis, data curation, methodology, writing – original draft. Sean Sullivan: investigation, formal analysis, data curation, methodology. Mackenzie Edinger: investigation, data curation. Brian Riley: validation, methodology, writing – review and editing. Krista Carlson: conceptualization, investigation, formal analysis, methodology, resources, supervision, funding acquisition, writing – original draft, writing – review and editing.

## Conflicts of interest

The authors declare no competing financial interests.

## Supplementary Material

RA-015-D5RA05518K-s001

## Data Availability

Specific datasets and raw files may be accessed for academic purposes upon reasonable request to the corresponding author. Supplementary information: Dynamic flow system setup, piping and instrumentation diagram and details about mercury generation are provided. See DOI: https://doi.org/10.1039/d5ra05518k.
