# Supplementary material for: Feasibility of carbon foam-based sorbents for the abatement of gaseous mercury and iodine
Source: RSC Adv. 2025 Sep 10;15(39):32708–20. doi: 10.1039/d5ra05518k (PMC12421304; doi:10.1039/d5ra05518k)
Supplement: RA-015-D5RA05518K-s001 [file RA-015-D5RA05518K-s001.pdf]

Supplementary Information (SI) for RSC Advances.
This journal is © The Royal Society of Chemistry 2025

Supporting Information

Feasibility of carbon foam-based sorbents for the abatement of gaseous mercury and iodine

*Karthikeyan Baskaran[a], Laurel Sharpless[a], Casey Elliott[a+], Sean Sullivan[a+], Mackenzie Edinger[a+], Brian Riley[b,a], Krista Carlson[a]**

[a] Chemical and Materials Engineering, University of Nevada, Reno, Reno, Nevada, 89557, United States

[b] Pacific Northwest National Laboratory, Richland, Washington, 99354, United States

* Corresponding author: Krista Carlson, E-mail: kc@unr.edu

+Affiliated with UNR during research

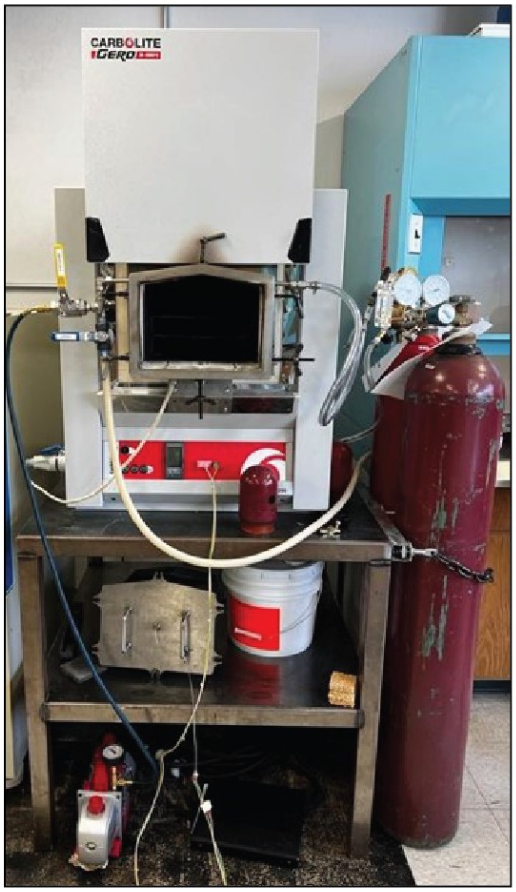

Figure S1. The furnace and retort used for carbonization of melamine foam to carbon foam (CF).

Table S1. Major components in LAW off gas.

**Component**	**Concentration (Vol %)**
**N_2**	69.3
**O_2**	18.6
**H_2O**	9.7
**CO_2**	1.01
**Ar**	0.83

Table S2. Minor components present in LAW off gas. VOC denotes volatile organic carbon.

Component	Concentration ($kg\ h^{-1}$)
**Hg**	2.69E-03
**NH_3**	2.69E-02
**NO**	1.29E+01
**N_2O**	3.44E+00
**NO_2**	1.72E+01
**CO**	9.25E-01
**H_2**	3.24E-02
**HCl**	4.40E-02
**HF**	2.39E-03
**^{129}I**	1.29E-04
**SO_2**	2.78E-02
**HNO_2**	2.45E-01
**HNO_3**	6.17E-02
**VOC**	4.88E-01
**Particulate**	1.08E-05

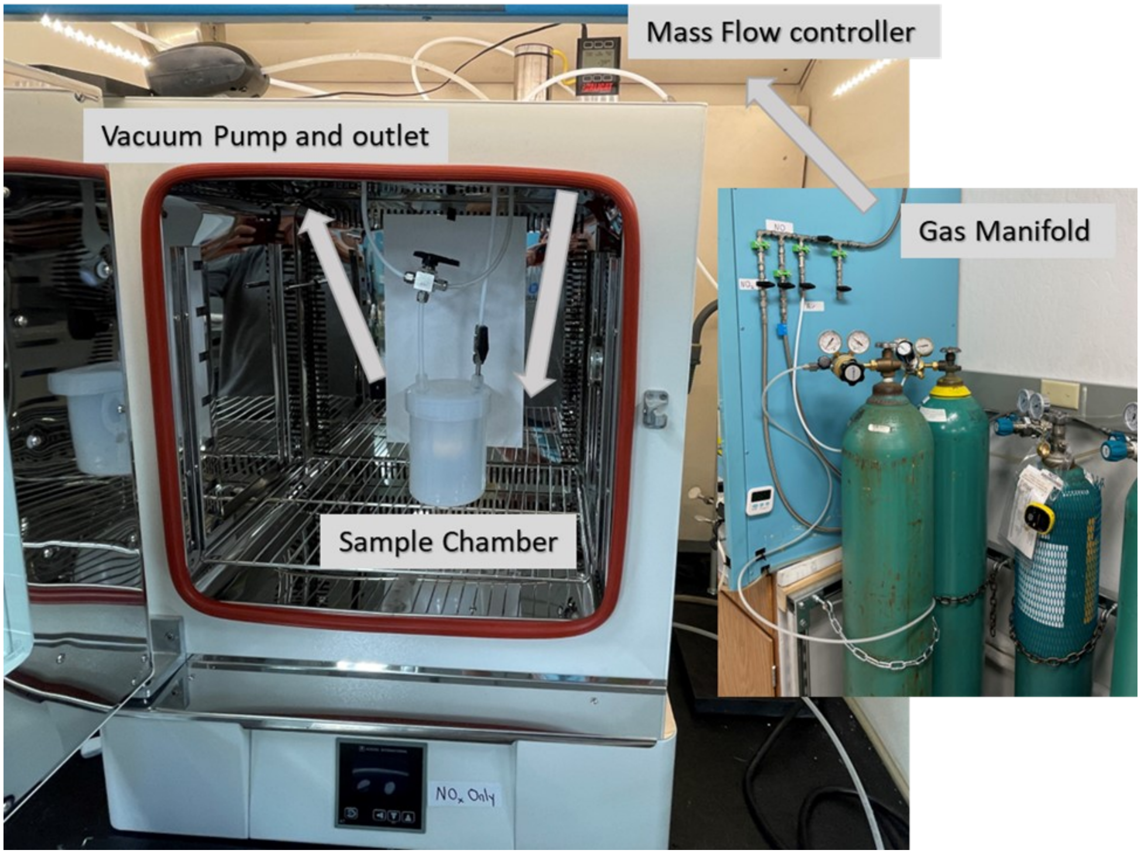

Figure S2. Setup used for NO_2 treatment, Sample was placed inside PFA jar and 1 v/v % NO_2 was flowed for 24 h at 150°C.

Static iodine loading

Samples were pre-dried under vacuum, weighed, and sealed in glass vials alongside control vials in a 1 L PFA container. Excess solid iodine was used to ensure a saturated iodine vapor environment. The sealed setup was placed in oven preheated to 150 ± 2°C for 24 hours, after which the vials were reheated in an uncovered container to remove any physisorbed iodine for 1 h and 24 h. The mass gain from iodine sorption was recorded and used to calculate both the iodine content and sorption capacity.

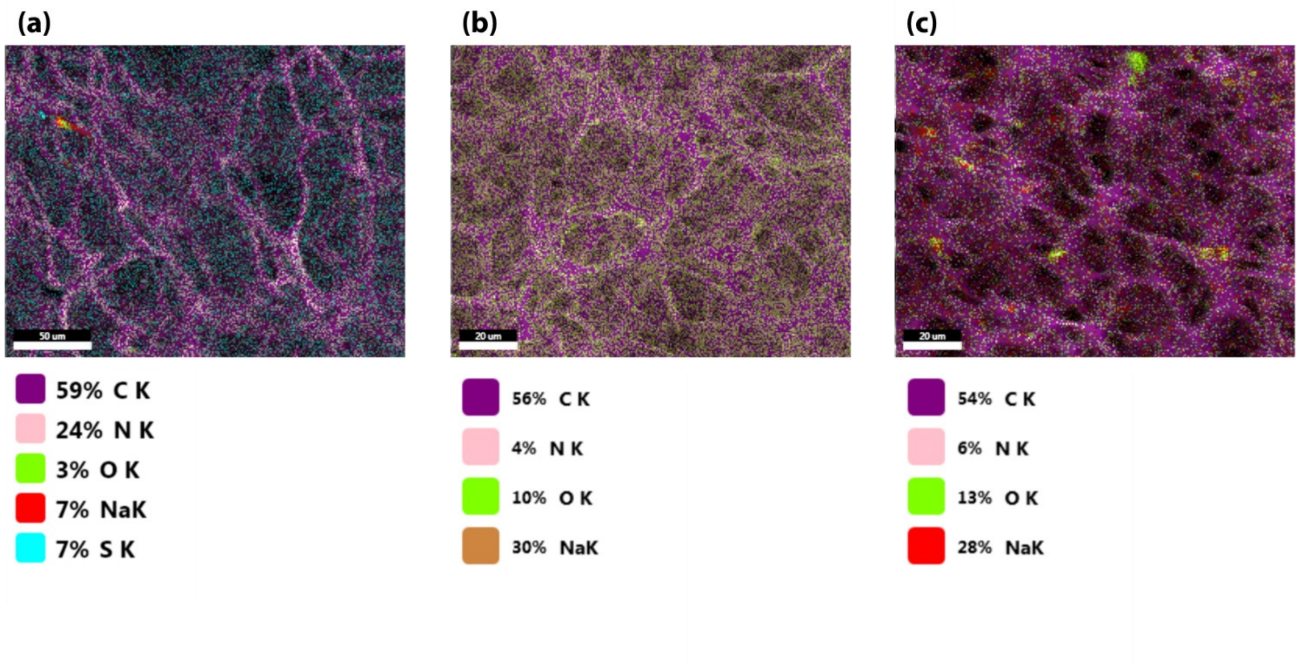

Figure S3. EDS map of (a) MeF, (b) CF, and (c) CF-NO_2. Changes in relative composition and absence of sulfur can be observed.

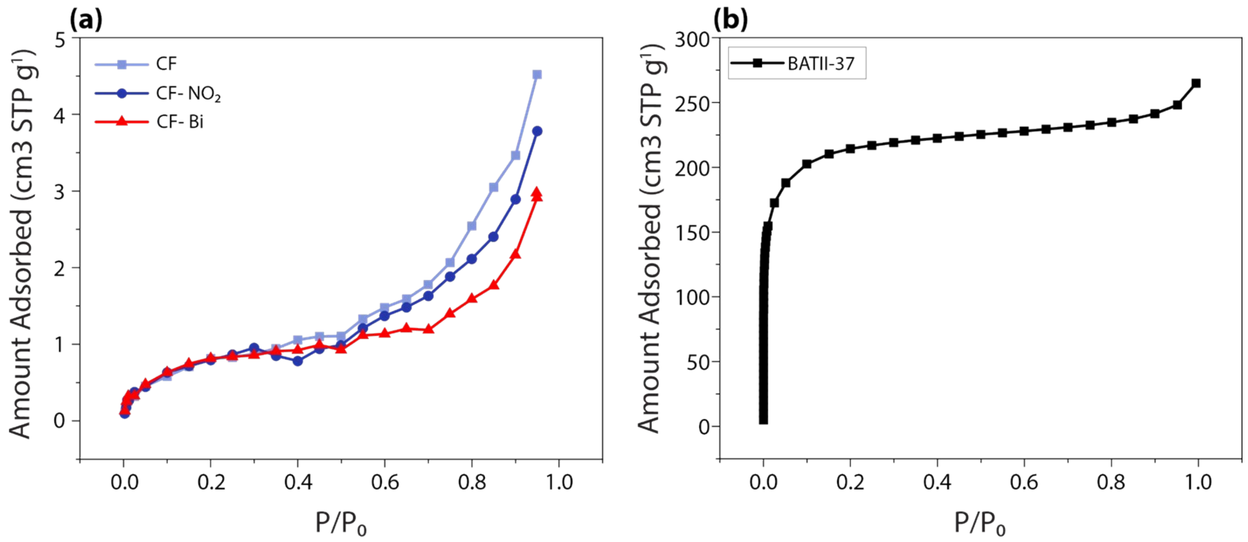

Figure S4. Argon adsorption isotherm of (a) CF sorbents including as prepared, CF-NO_2 and CF-Bi and (b) BATII-37

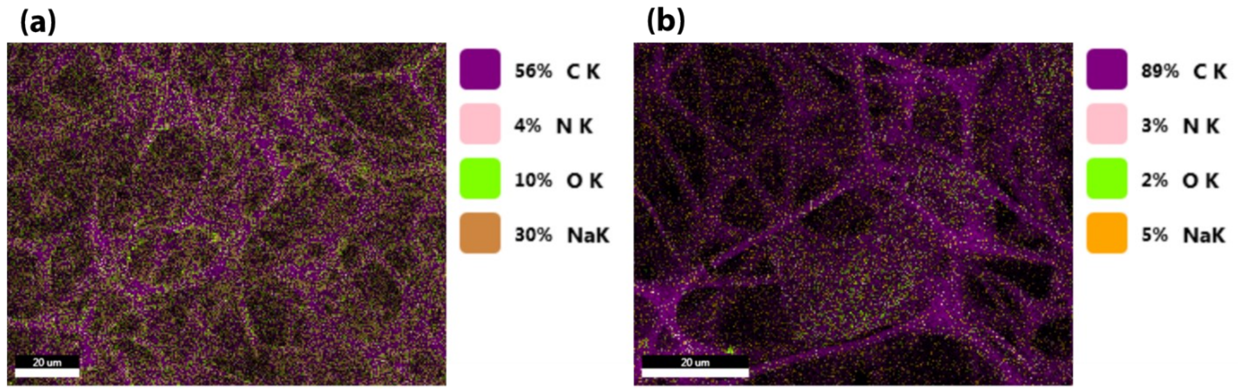

Figure S5. EDS mapping showing relative concentration of (a) CF and (b) CF after iodine loading and stripping with water. Iodine was not observed after soaking the foam in water.

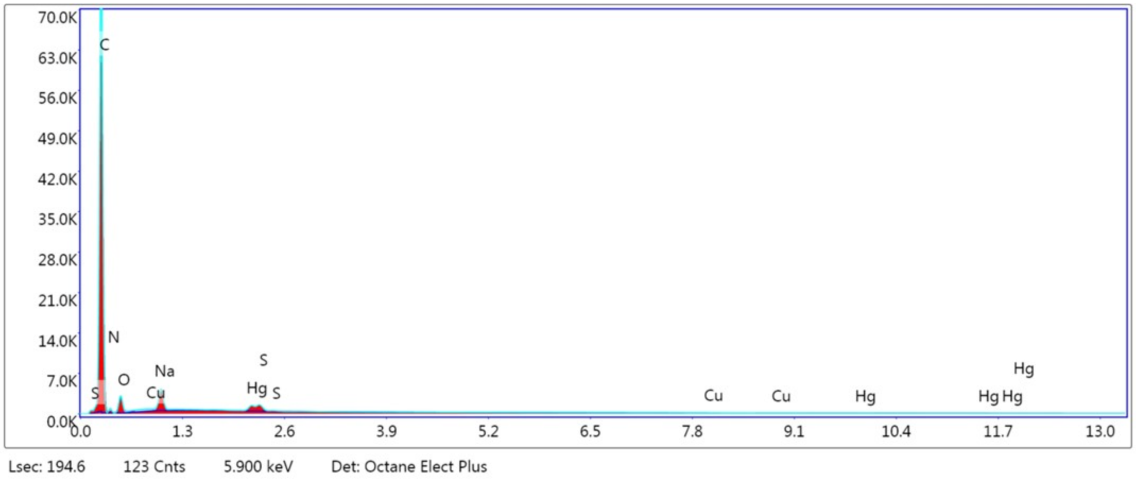

Figure S6. EDS spectrum of static mercury loaded CF.

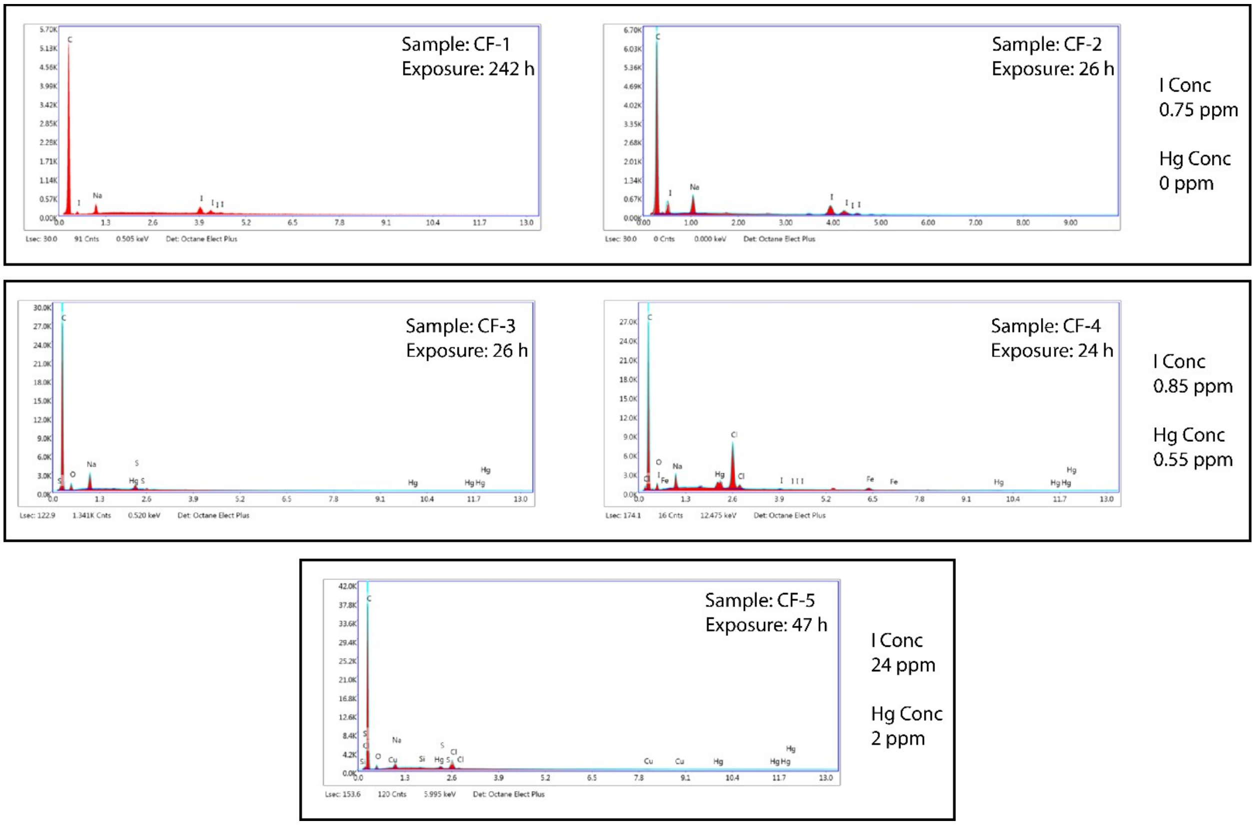

Figure S7. EDS spectra from dynamic testing with Hg and iodine vapors. Details of the sample, exposure time and source concentration are provided along with the spectrum.

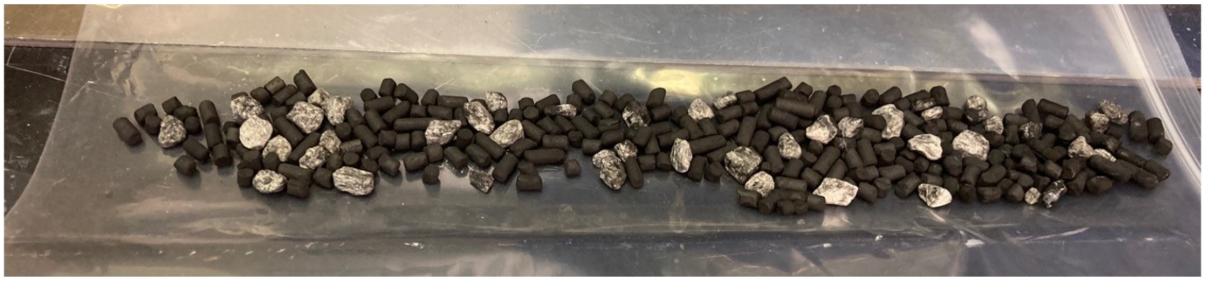

Figure S8. BATII-37 after exposure to iodine, mercury, and elevated moisture poured out of the bed for partitioning.

The V1 system

The V1 system (Figure S8) was designed to be modular to easily allow for infrastructure changes (e.g., various primary and guard sorbent bed sizes) and a range of gas mixtures. Primary and guard sorbent beds were in series and had dimensions of 3” length and 0.87” ID (30 cm^3 volume). Two sets of sorbent beds were arranged in parallel, and the range of face velocities from 10 ft min^{-1} to 68 ft min^{-1} gave residence times between 0.22 s and 3 s.

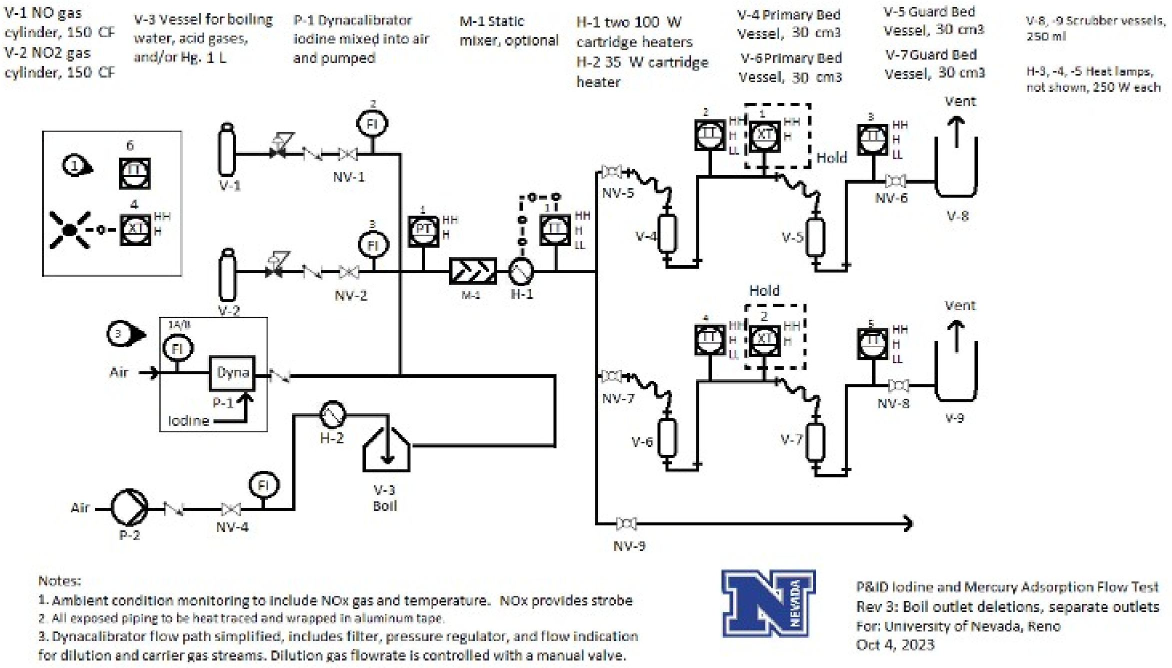

Figure S9. (a) P&ID of the V1 system. Gas mixing is shown on the left, sorbent beds are shown on the right.

Dynacalibrator

The Dynacalibrator (Dynacal, Model 230-28B-I, VICI Metronics Inc.) is a commercial product advertised to supply precise quantities of a species for a carrier gas. The desired species is contained in a permeation tube (a PFA sleeve) that releases the species through the walls of the tube. Figure S9 shows the internal plumbing of the Dynacal instrument. It should be noted that only the permeation chamber is heated, and many of the critical parts are not Teflon (e.g., the permeation chamber, differential pressure regulators), which can allow for reactions with and/or accumulation of mercury or iodine.

Permeation tubes (Hg and I_2, VICI) are sold with an advertised permeation rate at a given temperature. To obtain an accurate mass balance of a given species in the system, the amount introduced must be known. In this case, after installing a permeation tube with a given permeation rate, the concentration of the species leaving the Dynacal is calculated using Eqs. 10 and 11.

$$\text{Concentration (ppm)} = \frac{K * P}{F} \tag{10}$$

$$K = \frac{24.45}{MW} \tag{11}$$

K accounts for the grams of the species in the volume of gas; *P* is the permeation rate of the tube in ng min^{-1}, calculated by the manufacturer based on experimental calibration; *F* is the chamber carrier flow rate in mL min^{-1}; and *MW* is the molecular weight of the species in g mol^{-1}. It should be noted that this assumes ideality of the gas at standard ambient temperature and pressure (25˚C and 1 atm). If more conditions of the system are known, they can be accounted for by calculating a more accurate molar volume (instead of 24.45 L mol^{-1}).

A significant challenge related to our system is the single chamber for the permeation tubes. As a result, any potential reactions between mercury and iodine, which are likely to occur, must also be considered.

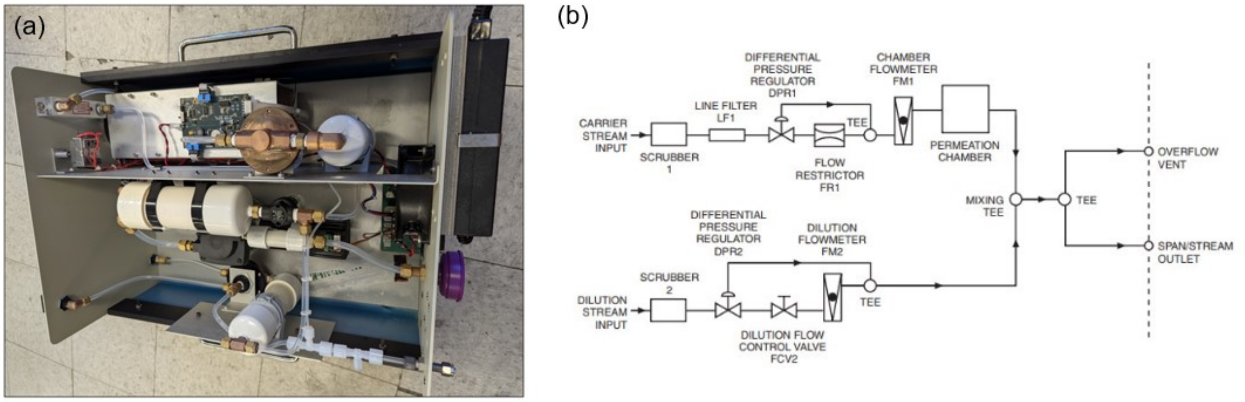

Figure S10 (a) Photo and (b) schematic of internal plumbing of Dynacal

Residual mercury and iodine in Dynacalibrator.

In addition to variability of the permeation tube, residual species in the system may also contribute to fluctuations in measured concentration. Therefore, bake-out testing was performed to assess the concentration of residual species remaining in the Dynacal following a run. A bake-out period is not specified by the manufacturer, but previous data from V1 and V2 testing indicate that residual mercury and iodine is left in the system after a 72-hour period.

Testing started with residual mercury from V2 testing that had completed in August 2024. The mercury permeation tube was removed from the Dynacal in early September, and the system was operated semi-continuously at 100°C with no permeation tubes until October 3rd, 2024 when an iodine permeation tube (7421 ng min^{-1}) was installed for preliminary iodine scrubber testing. Concerns that residual mercury may be remaining in the Dynacal prompted more intentional bake-outs.

To analyze the bake-outs, permeation tubes were removed from the Dynacal, and ambient air was pumped through the Dynacal. The effluent was collected in a concentrated nitric acid solution in

scrubber apparatus A. Periodic analyses of the scrubber solution by EPA Method 1631 showed significant quantities of residual mercury present even after hundreds of hours of Dynacal operation over 3 months (Figure S10).

The data presented in Figure S10 is normalized to the exact length of time the scrubber was connected to the Dynacal effluent (between 17 and 72 hours). With the V2 mercury permeation tube installed, the Dynacal was supposed to release 36 μg hr^{-1}. As the Dynacal is operated over time, the scrubber collects decreasing quantities of mercury. As a control, the cleaned scrubber apparatus A retained ~ 1 μg of mercury. The extent to which iodine was in contact with mercury from the V2 system and aided in the bakeout of the Dynacal is unclear.

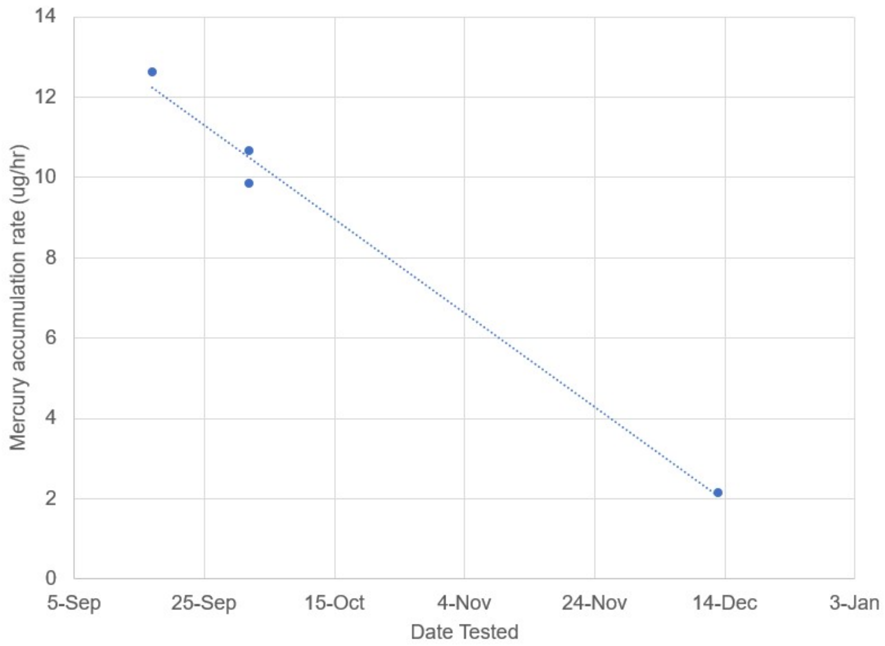

Figure S11. Residual mercury accumulation rate in acid scrubber over 3 months of semi-continuous Dynacal operation. No permeation tube was loaded for the bake-out tests.

Similar conclusions were drawn for iodine permeation tubes, where residual iodine was observed after switching to a serialized permeation tube with a lower rate (544 ng min^{-1} ± 0.79%). The operation of the Dynacal at 100 °C over the span of 1440 h is denoted by a Gantt chart in Figure 37a. Bake-outs were conducted in between experiments with the 544 ng min^{-1} permeation tube installed. The amount of iodine that was collected during some of these bakeouts and during these experiments are shown in Figure S11b.

Based on the results depicted in Figure S11, an extensive bakeout (at least 300 h) was needed after removing the 7421 ng min^{-1} permeation tube to approach the expected permeation rate of the newly installed tube (544 ng min^{-1}). While able to get somewhat consistent results once this was achieved (see Run 2-1 and Run 3-1), this makes the Dynacal unrealistic to use if the permeation tube is to be replaced with one having a different permeation rate or species.

Therefore, a substantial bake-out period is required to fully remove residual mercury or iodine from the Dynacal when changing the permeation tube. However, prolonged interactions between

these species and the system may introduce fluctuations in the concentrations data that are difficult to distinguish from sorbent behavior.

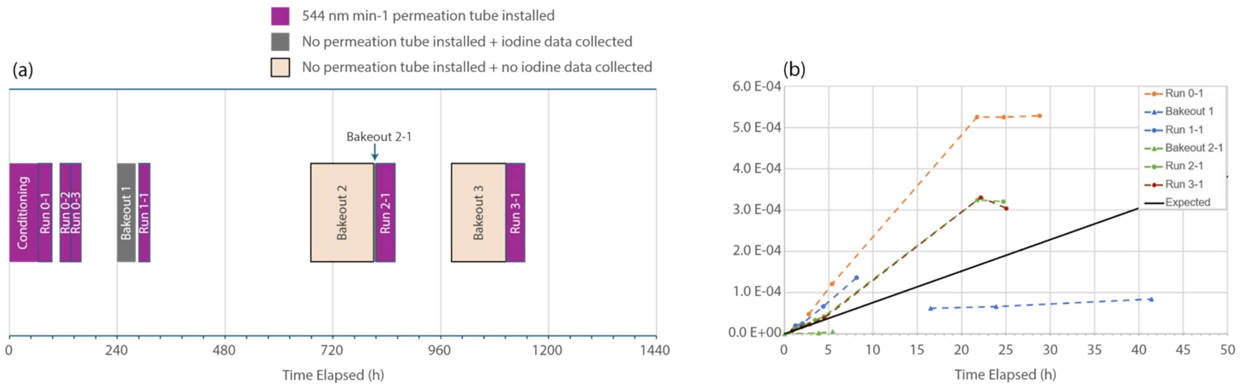

Figure S12. (a) Schedule, in hours, that the Dynacal was operated at 100 °C after removing the 7421 ng min^{-1} tube, with and without the 544 ng min^{-1} tube installed; (b) scrubbed iodine from the Dynacal, collected intermittently as denoted. “Bakeout” indicates that no permeation tube is installed.
